# Intranasal delivery: An attractive route for the administration of nucleic acid based therapeutics for CNS disorders

**DOI:** 10.3389/fphar.2022.974666

**Published:** 2022-08-30

**Authors:** Pranav Shah, Manisha Lalan, Kalyani Barve

**Affiliations:** ^1^ Maliba Pharmacy College, Uka Tarsadia University, Surat, India; ^2^ Sat Kaival College of Pharmacy, Sarsa, India; ^3^ Shobhaben Pratapbhai Patel School of Pharmacy and Technology Management, SVKM’s Narsee Monjee Institute of Management Studies, Mumbai, Maharashtra, India

**Keywords:** CNS disorders, drug delivery, nasal route, regulatory challenges, toxicity, nucleic acid, nucleic acid based therapeutics

## Abstract

The etiologies of several cardiovascular, inflammatory, neurological, hereditary disorders, cancer, and infectious diseases have implicated changes in the genetic set up or genetic mutations as the root cause. Nucleic acid based therapeutics (NBTs) is a new class of biologics that are known to regulate gene expression at the transcriptional and post-transcriptional level. The NBTs include oligonucleotides, nucleosides, antisense RNA, small interfering RNAs, micro RNA etc. In recent times, this new category of biologics has found enormous potential in the management of cardiovascular, inflammatory, neurological disorders, cancer, infectious diseases and organ transplantation. However, the delivery of NBTs is highly challenging in terms of target specificity (intracellular delivery), mononuclear phagocyte system uptake, stability and biodistribution. Additionally, management of the above mentioned disorders require regular and intrusive therapy making non-invasive routes preferable in comparison to invasive routes like parenteral. The nasal route is garnering focus in delivery of NBTs to the brain in the management of several CNS disorders due to the associated merits such as non-invasiveness, possibility of chronic delivery, improved patient compliance, avoidance of hepatic and gastrointestinal metabolism as well as ability to bypass the BBB. Hence in recent times, this route has been sought by the reserachers as an alternative to parenteral therapy for the delivery of several NBTs. This review shall focus on an array of NBTs delivered through nasal route, their challenges, applications and opportunities. The novel delivery systems for incorporating NBTs; their targeting strategies shall be critically reviewed. The challenges towards regulatory approvals and commercialization shall also be discussed at large. Comparison of learnings derived from the success and barriers in nasal delivery of NBTs will help in identification of futuristic opportunities for their translation from bench to bedside.

## 1 Introduction

### 1.1 Introduction to nucleic acid based therapeutics

Nucleic acid-based therapeutics (NBTs) implies the use of nucleic acid-based approaches that target gene expression by making changes to the DNA or RNA. These majorly inhibit the production of specific faulty proteins leaving the other proteins intact. As opposed to conventional treatments, which produced transient therapeutic effects, these therapies produce specific, long-lasting and at times permanent cures to the underlying disease problem (Kulkarni et al., 2021). The basic mechanism of action for different NBTs is based on complementary base pairing with endogenous DNA or RNA.

There are several NBTs available for use in different disorders. Table 1 describes the classification, structure and mechanism of the NBTs. This may be required to design a drug delivery technique after knowing the site of action of these NBTs.

**TABLE 1:** Structure and mechanism of NBTs.

Nucleic acid therapy	Structure	Mechanism
DNA therapeutics		
Antisense oligonucleotides (ASOs)	Short synthetic nucleic acids (8–50 base pairs)/segments of DNA. Since ASOs consist of only a few bases the term “oligo” is associated with them	Binds to pre-mRNA or mRNA, the complex either gets degraded by cellular RNase or endogenous RNA is blocked and thus, regulate protein synthesis Kulkarni et al. (2021)
DNA aptamers	Synthetic oligonucleotide having single strand similar to ASO with a high affinity to target based on structural conformation. The target can be a small molecule, protein or the cell.	They either inhibit the protein-protein interaction Kannan Sridharan et al. (2016) or promote the function of target protein Zhu et al. (2015)
Plasmid DNA	Double stranded DNA with a higher molecular weight	Upon entry into the cell, DNA transcription and translation generates a therapeutic protein Pushpendra et al. (2012)
DNAzymes	DNA molecule having single strand	They bind to their target mRNA by base pairing and cause cleavage in the RNA Chan and Khachigian (2009).
Gene therapy	Gene expression cassette comprising of a promoter region, transgene of interest and a terminator region	Replacement of abnormal or non-functional gene Kannan Sridharan and Gogtay, (2016)
RNA therapeutics		
*RNAi (RNA interference)*		
siRNA-small interfering RNA (synonyms: short interfering RNA, silencing RNA)	RNA having either single or double strands (21–23 nucleotides)	Gets incorporated in the RNA induced silencing complex (RISC) and causes silencing/degradation of one full complementary mRNA Winkle et al. (2021). This leads to RNA interference blocking the translation of protein. Very effective in diseases caused by abnormal expression or mutation
MicroRNA (abbreviated as miRNAs or miRs)	Small nucleotide (17–25), single stranded non coding RNA which function as gene regulators	Are responsible for regulating gene expression by binding to complementary sequences of target messenger RNA, causing translational suppression or mRNA degradation Target multiple genes in a single pathway and can down-regulate gene expression Pushpendra et al. (2012)
miRNA mimics	ASO with a sequence complementary (fully or partially) to endogenous miRNA.	Doesn’t allow RISC loading and functions as anti-miRNA Kannan Sridharan and Gogtay, (2016)
miRNA sponges	Artificially made transcripts with multiple miRNA binding sites	Bind and inhibits miRNA Kannan Sridharan and Gogtay, (2016)
miRNA masking ASO	8–10nucleotide (locked nucleic acids)LNAs	Binds to miRNA binding site in a specific gene
Short hairpin RNA (ShRNA)	Artificial RNA with a hairpin turn	Cleaved as a double stranded product and forms the RISC resulting into silencing of mRNA
Circular RNA	RNA structure with a covalent bond between the 3′ and 5′ end	Bind to RNA binding protein or ribonucleoprotein complexes thus inhibiting the mRNA. Kannan Sridharan and Gogtay, (2016)
*RNA aptamers*		
RNA aptamers	Single stranded nucleic acid chain, capable to interact with proteins	Inhibits the protein by binding them Kannan Sridharan and Gogtay, (2016)
RNA decoys	Similar to RNA aptamers	Bind to proteins that act as mRNA stabilizing element, thus causing the degradation of mRNA Kannan Sridharan and Gogtay, (2016)
*Antisense RNA(asRNA)*		
Antisense RNA	Short deoxyribonucleotides (19–23) representing unique DNA transript	Bind to specific region of mRNA and block gene expression of that region
Ribozymes/Catalytic RNA	Similar to antisense RNA with an enzymatic moiety which can cleave the RNA	Functions as an enzyme, catalyses the hydrolysis of phosphodiester bonds and causes the degradation of the target RNA Dönmüş (2021).
*Other RNA therapeutics*		
Long noncoding RNA (LncRNA)	Large nucleotide (more than 200), single stranded non coding RNA which function as gene regulators	Can interact with DNA, RNA and proteins and cause transcriptional post transcriptional inhibition of protein synthesis.
Heterogenous nuclear ribonucleoproteins (hnRNPs)	RNA binding proteins	Important for maturation of mRNA, thus modulating the expression of mRNA. Might be important in the development of cancer and neurodegenerative diseases. Geuens et al. (2016)

**FIGURE 1:**
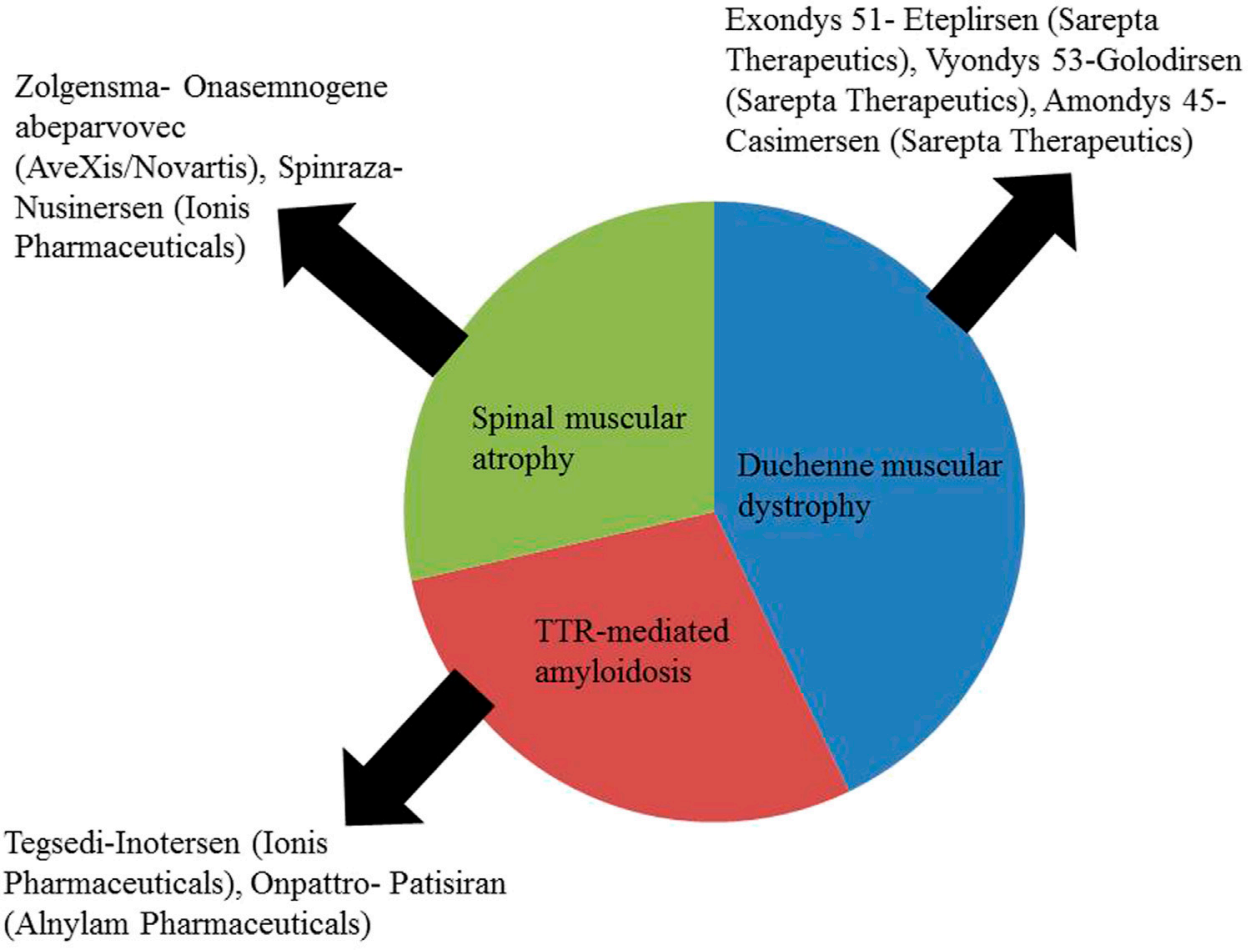
Approved NBTs for the treatment of CNS disorders (Kulkarni et al., 2021).

NBT is a relatively new concept and a few have been approved by the licensing authorities (Figure 1)

These NBTs are given either by intrathecal, subcutaneous or intravenous route but none is available as an intranasal preparation. There is a scope for developing an intranasal formulation of these approved NBTs.

### 1.2 Therapeutic uses of Nucleic acid based therapeutics

The NBTs as described earlier is used to treat as well as diagnose a variety of CNS disorders, the major ones are briefly discussed below:

#### 1.2.1 Neurodegenerative disorders

Parkinson’s disease is characterized by progressive loss of dopaminergic neurons leading to movement disorders. Multiple genes such as α-synuclein, LRRK2, PARK7, PINK1, and PRKN are involved in this disorder. ASOs targeting SNCA, LRRK2, and improving dopamine transmission are underway. ASOs that restore the parkin protein, gene therapy to induce enzymes required for dopamine biosynthesis or reduce the activity of dopamine degrading enzyme, siRNA which reduce SNCA mRNA levels, short hairpin RNAs that knockdown α-synuclein, miRNA-7, miRNA-153, miRNA-214; which in turn downregulate α-synculein expression and aptamers selectively binding to α-synuclein are some other NBTs for therapeutic management of Parkinson’s. Aptamers in this case, also called as apta-sensors, can also be used as diagnostic and monitoring markers since they can determine the concentration of dopamine (Li et al., 2020).

Alzheimer’s disease (AD) is another neurodegenerative disorder caused due to loss of cholinergic neurons leading to learning and memory deficit. Mutations in various genes such APP, PSEN1, PSEN2 and mutations in tau protein are major causes and risk factors for AD. ASOs targeting mutated tau proteins, ASOs increasing the expression of BDNF, other ASOs removing the amyloid plaque, and gene therapy to decrease the expression of APOE4 are currently under different investigative stages for the treatment of AD. Apart from having therapeutic importance, miRNAs (a panel of 12 miRNAs) related to AD can also be used as diagnostic biomarkers as they are amply available in blood, CSF, plasma and serum (Walgrave et al., 2021).

To treat temporal lobe epilepsy, which fails to respond to conventional anti-seizure medications in at least 30% of the cases, miRNA and small non coding RNA could be the targets of ASOs and antimiRNAs and can be used to suppress a set of proteins responsible for the seizures (Morris et al., 2021). miRNAs are also being projected as targets for CNS injury like traumatic brain injury, spinal cord injury or stroke. miRNA mimics or antagonists to specific miRNA are proposed as therapies for treating CNS injuries. The same miRNAs can also be used as diagnostic biomarkers for such injuries (Sun et al., 2018).

#### 1.2.2 Psychological disorders

Approximately 12% of people worldwide suffer from mental disorders which disturb the thinking and behavior of a person and lead to poor quality of life. Novel therapeutics are needed for the treatment of anxiety disorders for the obvious reason of higher efficacy and less adverse effects. miRNA may play a role in the development of anxiety disorders and can be used as biomarkers for diagnosis or as target for the treatment of these disorders, though such research is in a budding stage (Scott et al., 2015). Depression, another common psychiatric condition, involves circRNAs namely circDYM and circHIPK2 in the modulation of depression mainly by serving as regulators of autophagic processes. Either knockdown or over-expression of these circRNAs might help in treating depressive symptoms (Gan et al., 2021).

#### 1.2.3 Cancer/tumor

Dysregulation of small non-coding RNAs and miRNAs has been implicated in the progression of cancer. Every molecularly different cancer cell will have a specific miRNA profile. Accordingly, these can be used as diagnostic biomarkers for a specific type of cancer (Mishra et al., 2016).

Dysregulation in miRNA can lead to alterations in the genes linked to cancer progression; the process of apoptosis and angiogenesis is controlled by miRNAs. They are also implicated in tumor immunity, tumor progression, metastasis, and multidrug resistance. Apart from miRNA, long non-coding RNA, circular RNAs, and small nuclear RNAs participate in miRNA monitored tumor regulation. As miRNAs participate in cancer development, they are also targets in cancer therapeutics (He et al., 2020). miRNA therapy as replacement therapy is usually preferred in combination with chemotherapy, radiotherapy, or immunotherapy to improve tumor killing/cancer elimination response.

miRNA-based vaccines which serve as gene expression units, RNA interference using siRNA or miRNA, and adjuvant effect using CpGODN, nucleic acid-based TLR agonist are other NBTs available for cancer therapy (Hager et al., 2020).

#### 1.2.4 Orphan diseases/Neurological disorders

Orphan diseases that have a known genetic cause are comparatively easier to treat with NBTs (Khorkova et al., 2021). Spinal muscular atrophy caused by the reduced levels of SMN1 protein can be treated with gene replacement therapy onasemnogene abeparvosec. Familial amyloid polyneuropathy caused due to excess deposition of transthyretin (TTR) in CNS and peripheral organs can be treated with Inotersen- an ASO and Patisiran-anti-TTR siRNA. Amylotropic lateral sclerosis is characterized by neuronal degeneration and severe muscle dystrophy. ASOs are being developed to target the mutated enzymes and proteins and either suppress them or cause their degeneration. RNA interference, CRISPR are underway for those with mutations of SOD1 gene, expansion of ATXN2 trinucleotide, repeat expansions of C9orf72 hexanucleotide, and FUS mutations (Amado and Davidson, 2021; Dönmüş 2021). Huntington’s disease is caused due to CAG repeat sequence in Huntington’s gene. ASOs and siRNA designed to knockdown this gene and reduce the expression of the toxic HTT protein are under investigation. Delivery of these NBTs at the cortex and striatum is a problem, however, scientists are working to find options for site-specific delivery (Zhang et al., 2021) (Khorkova et al., 2021).

Neuronal ceroid lipofuscinoses (NCL or CLN) collectively known as Battons disease is characterized by the accumulation of an autofluorescent lipopigment leading to neurodegeneration. Gene therapy and other NBTS are under investigation for various type of CLN gene mutations.

Epilepsy is characterized by recurrent seizures and is a chronic condition. Currently used anti-seizure medications simply treat the symptoms and not the underlying cause. It has been noted in animal experiments that miRNA can modify epileptogenesis (Feng et al., 2020). These miRNAs function as regulators of seizure-induced neuronal death and also influence the innate immune response of the astrocytes (Liu et al., 2013). Dravets syndrome is caused due to mutations in the SCN1A gene causing a severe form of childhood epilepsy and sudden death. This gene is responsible for the formation of voltage-gated sodium channels. SCN1A upregulation can be achieved with the use of NBTs such as mRNAs and ASOs.

Mucopolysaccharoidosis is another orphan disease characterized by abnormalities in mucopolysaccharide metabolism, due to mutations in related genes. It may lead to various forms of developmental abnormalities or intellectual disabilities. Angelman syndrome is characterized by a deficiency of the gene UBE3A leading to late development, ataxia, and seizures. Canavan disease caused due to mutations in ASPA gene leading to the accumulation of N-acetylaspartate leading to severe psychomotor impairment and early death. Friedrichs ataxia, another neurodegenerative disease caused due to expanded AAG sequence in the Frataxin gene leads to progressive loss of movement and sensations. Gene therapy and miRNA-based treatment are currently under investigation for all the above mentioned disorders.

Aptamers are under investigation to enhance the efficacy of thrombolytic treatment-the mainstay for stroke management. ASOs have also shown promising results in treating stroke specifically of the genetic origin. miRNAs that regulate reperfusion-induced neuronal injury can also be targeted for preventing stroke injury (Henninger and Mayasi, 2019). Circular RNA SCMH1 has been identified to regulate the expression of genes that maintain brain function and are found to be depleted in individuals post-stroke. A study has proved that administration of this circRNA significantly enhances post-stroke recovery (Yang et al., 2020).

**FIGURE 2:**
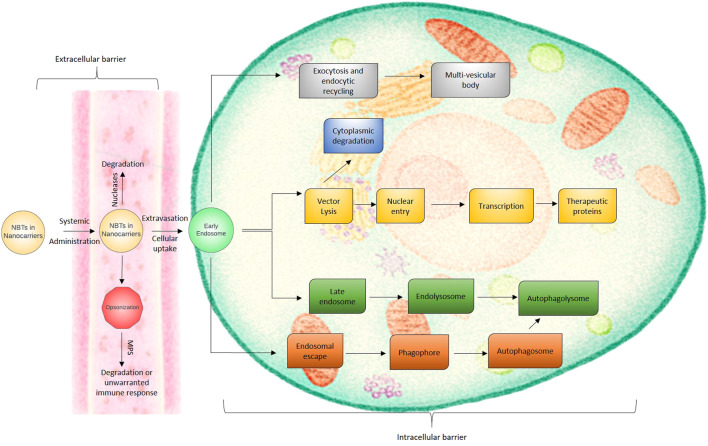
The extracellular and intracellular biological barriers for nanocarrier based NBT delivery.

Though NBTs may offer options for the treatment of various disorders as mentioned above and can also be converted as a tailor made therapy for the individual patient, it still has some limitations and barriers. The barriers are extracellular and intracellular as shown in Figure 2. The extracellular barriers include clearance through the kidney, liver and spleen, susceptibility to nucleases, and extravasation. The intracellular barrier majorly includes passage across the cell membrane to ensure cellular entry and further delivery into the nucleus. As mentioned in the earlier sections, though the therapy might be very effective, it is a challenge to deliver it inside the target cell. NBTs might activate the immune system, might produce off target effects, and may lead to systemic accumulation. Due to the possibility of delayed onset of side effects, high vigilance is required for such therapies. Moreover, clinical translation of such therapeutics is a challenge because firstly many times the animal study is performed using healthy animals and secondly the half-life of nucleotides is very short (Landmesser et al., 2020). Many scientists are working to overcome these limitations. Modifications of the structure, conjugation with other molecules, and targeted drug delivery approach are some of the areas which have shown a promising solution to the challenges mentioned above. Delivery of nucleotides to the site of action is affected by multiple determinants. One of the major factors controlling the success of this endeavor is the route of delivery or administration. In this review, we shall discuss the delivery approach for NBTs to the brain *via* the nasal route so that they can target the brain areas and exhibit their effect in the treatment of CNS-related disorders.

### 1.3 Nasal delivery for Nucleic acid based therapeutics

**FIGURE 3:**
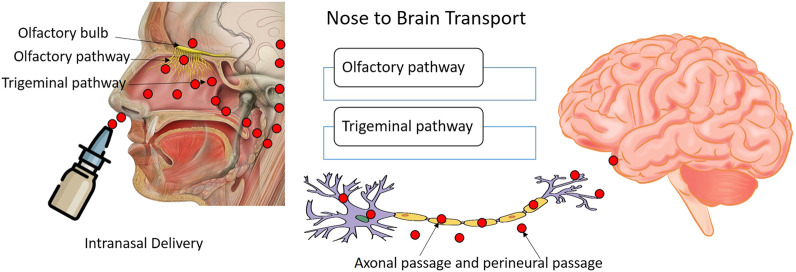
Intranasal administration of drugs to reach brain along the olfactory, trigeminal nerves.

The primary reason for the attention to the nasal route of delivery of nucleotide drugs is the possibility of obviating the BBB and allowing direct access to the CNS. The BBB is a formidable barrier that restricts the entry of macromolecules like nucleotides very efficiently. Nose to CNS delivery can show rapid transport of the decoy along the olfactory and trigeminal neural pathways (Figure 3). The trafficking pathways for the payload may be extracellular or intracellular. The extracellular pathway is faster and can be observed along the perineural channels of the nerve cells to access the CNS post its entry into the paracellular space of the nasal epithelium. The intracellular pathway encompasses the endocytosis of the decoy by olfactory sensory cells and passage through the axons to the synaptic clefts present in the olfactory bulb region followed by exocytosis. The transsynaptic transport process is repetitive which helps in distributing the payload to other brain regions. The intravenous administration of nanocarrier-based nucleotide delivery is prone to rapid systemic clearance through the reticulo endothelial system and poses issues in breaching the BBB (blood-brain barrier). Nose-to-brain pathways not only permit quick access to CNS, but the nucleic payload can persist in the CNS for long durations of time suggesting the plausibility of prolonged therapeutic or pharmacodynamics responses. The investigations on nasal delivery of nucleotide drugs have revealed that nucleotide delivery to different regions of the brain and multiple cell types can be achieved through this route. Studies suggest the dominance of the olfactory route for the NBT trafficking. However, characteristics like hydrophilicity, presence of tight junction permeabilizers, and choice of nanocarrier may influence the dominant pathway. Further, studies have suggested that site-specific delivery of nucleotide drugs tends to minimize the off-target toxicities and effects. The minimal systemic exposure of these potent agents ensures that other organs like the liver, spleen, and kidney are protected from exposure. Minimal off-target cellular permeation will translate to lower toxicities. The challenge for intranasal delivery is to ensure maximum CNS penetration and minimal exposure to the lungs as intranasal delivery leads to pulmonary ingress to some extent. Surface modifications of the nanocarriers have been investigated for targeted delivery to CNS through the nasal route. However, the nasal ciliary clearance and enzymatic activity in the tract limit the therapeutic potential of nasally administered nucleotide drugs. The unmodified nucleic acid drugs are particularly susceptible to cleavage by nucleases or triggering an immune response. Further, low-dose administration is only feasible with nasal delivery mandating frequent instillation. The chances of variable dosing may arise due to improper administration. The inter-individual differences in physiology and pathologies like cold and congestion can also introduce variability in absorption profiles (Podolska et al., 2012; Kanazawa, 2015; Dhas. et al., 2021; Hammond et al., 2021). Despite these limitations, the anatomical merits like enormous absorptive surface area, extensive vascularization, porous endothelial basement membrane, and bypassing the first pass hepatic biotransformation can lend beneficence to this route of delivery. Ease of accessibility, non-invasiveness, and self-administration mode makes nasal delivery particularly attractive from the clinical perspective. Nanocarrier encapsulation of NBTs, chemical modification can help offset the challenges faced by NBTs. Research focusing on enhancing the endosomal escape efficiency will permit higher cytosolic concentrations of NBTs. Thus, nasal delivery of nucleotides is practically feasible and attractive.

## 2 Application of nasal delivery of Nucleic acid based therapeutics in CNS disorders

### 2.1 silencing RNA

siRNAs have been employed in the management of several genetically influenced disorders (Neumeier and Meister, 2020). One of the major therapeutic segments, wherein siRNA-based therapeutics have been employed include CNS disorders, such as brain tumors, Alzheimer’s disease, and Parkinson’s disease (Mathupala, 2009).

Kanazawa et al. (2013) developed nanomicelles formulated using polyethylene glycol Polycaprolactone copolymers conjugated with cell-penetrating peptide-Tat (mPEG-PCL-Tat/siRNA nanomicelles). The nanomicelles were administered intranasally and their ability to deliver siRNA was evaluated. mPEG-PCL-Tat/siRNA nanomicelles exhibited a size range of 50–100 nm. The levels of siRNA transferred to the brain when naked siRNA (administered intranasally), mPEG-PCL-Tat/siRNA nanomicelles (administered intravenously), and mPEG-PCL-Tat/siRNA nanomicelles (administered intranasally) were found to be ∼0.2 % ID/g of brain tissue, 0.5 % ID/g of brain tissue and 2.0 % ID/g of brain tissue, respectively. Naked siRNA and mPEG-PCL-Tat/siRNA complexes were administered intranasally to Sprague-Dawley male rats and their biodistribution in brain tissue was studied. The results indicated the role of mPEG-PCL-Tat in delivering higher amounts of siRNA to the brain by noninvasive i.n. route *via* the trigeminal and olfactory nerve pathways. The authors suggest that the findings of this study might be extrapolated towards the treatment of intractable neuropsychiatric disorders, brain tumors, and cerebral infarction.

In a study by Kanazawa (2015), two formulations were prepared: (i) siRNA with a Raf-1 (siRaf-1) was loaded into mPEG-PCL-Tat micelles [mPEG-PCL-Tat/siRaf-1 complexes] and (ii) siRNA with a Raf-1 (siRaf-1) and camptothecin (CPT) were co-loaded into mPEG-PCL-Tat micelles [CPT-loaded mPEG-PCL-Tat/siRNA complexes]. These formulations were administered intranasally to investigate their therapeutic effects on a rodent model of malignant glioma. The particle sizes of mPEG-PCL-Tat/siRaf-1 complexes and CPT-loaded mPEG-PCL-Tat/siRNA complexes were 60–130 and 60 nm–200 nm, respectively. The *in vitro* cytotoxicities of the formulations were evaluated in rat glioma C6 cells wherein CPT-loaded mPEG-PCL-Tat/siRaf-1 complex exhibited a significant increment in the induction of cell death as compared to a combination of mPEG-PCL-Tat/siRaf-1 complex and CPT-loaded mPEG-PCL-Ta. The *in vivo* distribution studies confirmed the significantly higher concentrations of siRNA in the brain when administered *via* a non-invasive intranasal route as compared to intravenous injection. The mean survival time of untreated rats, rats treated with naked siRaf-1 solution, rats treated with mPEG-PCL-Tat/siRaf-1, rats treated with CPT-loaded mPEG-PCL-Tat/siControl, and rats treated with CPT-loaded mPEG-PCL-Tat/siRaf-1 were found to be 16.6, 18.4, 20.4, 20.6, and 28.4 days, respectively. The authors concluded that the cell-penetrating peptide-modified block copolymer accelerates the delivery of siRNA/drug to the brain *via* the nose, and proposed the use of this drug in the clinical therapy of brain tumors and CNS disorders.

Kim et al. (2012) investigated the therapeutic effectiveness of siRNA in the postischemic rat brain upon intranasal administration. e-PAM-R, a biodegradable PAMAM dendrimer, was employed as a gene carrier. The animals received three different treatments namely: high mobility group box 1 (HMGB1) siRNA, FITC-labelled transfection indicator siRNA, and nonspecific siRNA. The study demonstrated the ability of the dendrimer to successfully deliver siRNA, which in turn led to the subsequent target gene knockdown in various regions of the brain such as striatum, amygdala, cerebral cortex, and hypothalamus. Additionally, the nose-to-brain delivery of HMGB1 siRNA led to significant alleviation of neurological and behavioral deficits as well as a significant reduction in the infarct volumes [maximal reduction to 42.8% ± 5.6% at 48 h after 60 min of middle cerebral artery occlusion (MCAO)] in the postischemic rat brain. This study was amongst the first ones to add siRNA to the list of neuroprotectants that can be delivered intranasally.

Rodriguez et al. (2018) employed cationic linear polyethylenimines (PEI) as a carrier for siRNA against the Beclin1 gene. The developed nanoplexes (PEI-siBeclin1 nanoplexes) were administered by the non-invasive nasal route in the mice model and the siBeclin1 bio-distribution and efficacy were evaluated. The possible toxicity in different regions of the brain and target gene silencing efficiency was also evaluated. Nose-to-brain delivery of the nanoplexes resulted in the presence of siRNA in the glial cells of the prefrontal cortex and cytoplasm of neurons at 4 h and 24 h post-delivery, with no major adverse immune reaction being recorded. The studies exhibited significant downregulation of protein expression by 65% after 24 h and 43% after 48 h in brain tissues without any evidence of toxicity. Based on the findings, the authors concluded the potential of intranasal drug delivery in directly delivering the nanocomplex to the CNS, which could potentially offer an efficient means of gene silencing-mediated therapy in the HIV-infected brain.

Das et al. (2018) investigated the role of Mac-1 siRNA delivered by the noninvasive nasal route to hypoxic mice brain. Upon intranasal administration of Mac-1 siRNA to BALB/c mice, the siRNA was able to bypass the BBB and led to target gene knockdown in the hippocampus and prefrontal cortex regions of the brain. In addition, Mac-1 siRNA caused an increment in the neuronal viability, switching the microglial phenotype, reduction in inflammatory mediators, and alleviation of working memory impairment in the hypoxic brain. Treatment with Mac-1 siRNA for 3 times during continuous hypoxic exposure significantly downregulated the expression of TNF-α [F (1, 8) = 113.0, *p* < 0.0001], IL-1β [F(1, 8) = 60.42, *p* < 0.0001] and IL-6 [F (1, 8) = 289.8, *p* < 0.0001] level within the prefrontal cortex region. Thus, the authors concluded the potential role of Mac-1 siRNA as a novel therapeutic agent in the management of working memory impairment. Furthermore, suppression of Mac-1 receptor by non-invasive nasal delivery of siRNA might open newer and more effective therapeutic options for the treatment of other glial cell-induced neurodegenerative disorders.

Yang et al. (2022) developed multifunctional core-shell structure nanomicelles (HA/DP7-C) using cell-penetrating peptide (DP7-C) enveloped with hyaluronic acid (HA). siRNA was encapsulated within the developed nanomicelles and was administered intranasally to the rats to evaluate its potential in glioma. *In vitro* studies of the nanomicelles exhibited high cell uptake efficiency and low cytotoxicity. *In vivo* studies exhibited that intranasal administration of the developed HA/DP7-C could deliver the siRNA to the CNS through the trigeminal nerve pathway within hours. Additionally, increased accumulation was recorded at the tumor site, which could be explained due to the interaction between HA and CD44. The successful delivery of an antiglioma siRNA at the intracellular site in GL261 tumor-bearing mice led to inhibition of tumor growth, prolonged the survival time, and led to a decrement in the tumor volume. Additionally, toxicity tests were performed on rats, which exhibited no adverse effects on the trigeminal nerves and nasal mucosa. Thus, the authors concluded the role of HA/DP7-C as a potential delivery system that could be administered *via* the intranasal route for delivering siRNAs in glioma therapy.

### 2.2 Antisense oligonucleotides

**FIGURE 4:**
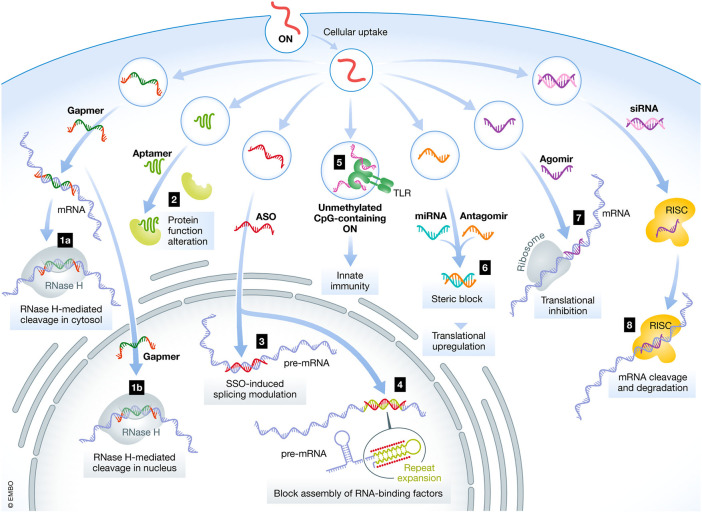
Mechanisms and location of action for various oligonucleotides. ON- Oligonucleotides, ASO- Antisense oligonucleotides, siRNA—small interfering RNA, mRNA- messenger RNA, miRNA–microRNA (Hammond et al., 2021 under Creative Commons Attribution License CC- BY).

In recent times, ASOs have gained great popularity in treating several diseases including neurodegenerative and neuromuscular disorders (Wurster and Ludolph, 2018). However, the clinical success of ASOs is hampered by rapid clearance and high susceptibility to nucleases (Dagle et al., 1991). Figure 4 highlights the various mechanisms and locations of action for oligonucleotides. The figure illustrates the intracellular and nuclear translocation of different NBTs. The parenteral route has been conventionally considered for delivery of ASOs but is associated with demerits such as invasiveness, risk of embolism and infection, cost of therapy, risk of hypersensitivity, and need for clinician for administration (Geary et al., 2015). The nasal route has received attention in recent times as an alternative route due to the associated merits of non-invasiveness, patient compliance, and avoidance of hepatic first-pass metabolism. However, the nasal delivery of ASOs is associated with limited bioabsorption and poor penetration into the cells. Researchers have employed the following strategies for overcoming the barriers in nasal delivery of ASOs: (a) chemical modifications wherein the phosphodiester backbone is replaced with a raised nuclease-resistant phosphorothioate backbone (b) development of delivery systems to extrinsically protect the oligonucleotides from metabolism by enzymes and (c) employing multifunctional polymers that exhibit permeation enhancement, enzyme inhibition and mucoadhesive properties (Vetter and Bernkop-Schnürch, 2010; Vetter et al., 2010; Borgonetti and Galeotti, 2021).

**FIGURE 5:**
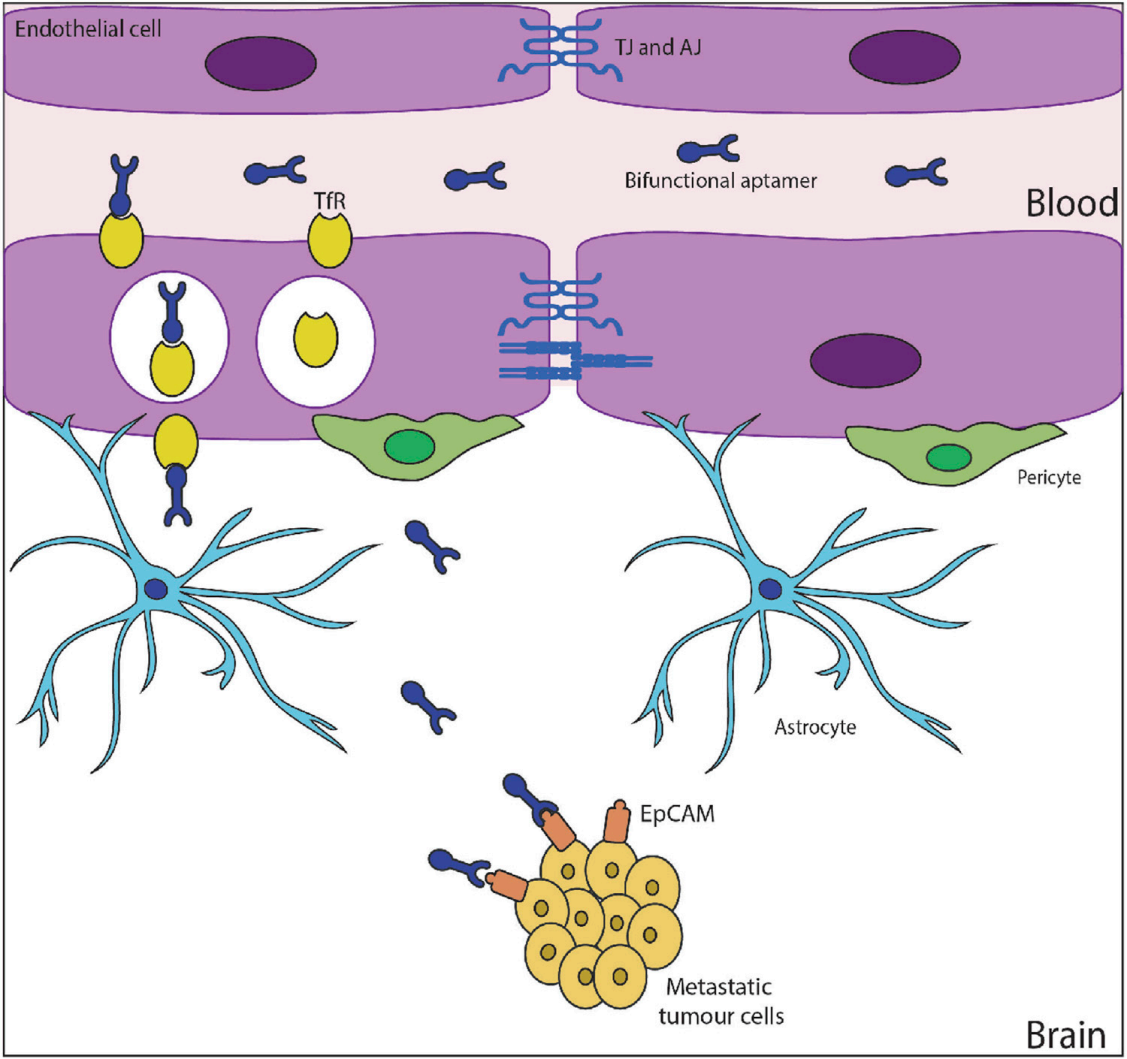
Apatmer transcytosis and release into brain microenvironment (Macdonald et al., 2018) under Creative Commons Attribution Non-Commercial License (http://creativecommons.org/licenses/by-nc/4.0).

Vetter et al. (2010) developed nasal mucoadhesive microparticles for the delivery of phosphorothioate antisense oligonucleotides (PTO-ODNs) employing the emulsification solvent evaporation technique. PTO-ODN microparticles were coated with either the mucoadhesive polymer polycarbophil-cysteine (PCP-Cys) or unmodified PCP and reduced glutathione (GSH). The microparticles were spherical shaped with a diameter of 30 µm and embarked on the advantages of reduced clearance rate from the nasal cavity, prolonged contact time with the nasal mucosa, high stability, improved permeation of ASOs, and controlled release. PTO-ODN incubated in thiolated polycarbophil/reduced GSH microparticles exhibited a 2.2-fold improved uptake from the nasal mucosa suggesting that thiolated polycarbophil/reduced glutathione microparticles shall be a promising carrier for intranasal delivery of phosphorothioate ASOs.

In another study, (Vetter and Bernkop-Schnürch, 2010) evaluated the role of thiolated polycarbophil as a multifunctional adjuvant in nasal delivery of phosphorothioate ASOs. In the presence of 0.45% thiolated polycarbophil and 0.5% glutathione, the uptake of phosphorothioate ASOs from the nasal mucosa was enhanced by 1.7-fold and the degradation was lowered. Thus, the studies confirmed the role of this novel polymer in providing protection towards mucosal-bound enzymes and enhancing the transport across porcine nasal mucosa without affecting the morphology of the mucosa.

Hashizume et al. (2008) administered GRN163 (an oligonucleotide N3’➝P5’thio-phosphoramidate telomerase inhibitor) to rats bearing intracerebral human tumor xenografts *via* the intranasal route. The findings of the *in vivo* studies indicated efficient distribution into an intracerebral tumor; inhibition of tumor growth, thereby prolonging the survival of athymic rats with no apparent toxicity. Administering GRN163, daily for 12 days *via* the intranasal route led to a significant prolongation of the median survival from 35 days in the control group to 75.5 days in the treatment group. The results of this study supported the futuristic role of intranasal GRN163 as a potential therapeutic agent for brain tumor patients and probably as a means for treating multifocal brain tumors and/or pediatric brainstem tumors, which otherwise are less amenable to potentially risky surgical procedures. In line with this investigation, nose-to-brain delivery of other tumor-specific agents could be studied for treating intracranial neoplasms.

Alarcón-Arís et al. (2018) performed the conjugation of inhibitory oligonucleotides- silencing RNA (siRNA) and antisense oligonucleotide (ASO), with the triple monoamine reuptake inhibitor indatraline (IND). IND-conjugated 499-siRNA (IND-499-siRNA) or IND-1233-ASO were administered to Wild-type male C57BL/6J mice *via* intranasal route at 30 μg/day for 4 consecutive days. IND-1233-ASO treatment selectively reduced endogenous α-synuclein mRNA expression in monoaminergic nuclei at 1-day post-administration to the extent of 84% ± 2% in substantia nigra pars compacta/ventral tegmental area; 70% ± 5% in dorsal raphe nucleus; and 79% ± 2% in locus coeruleus as compared to vehicle-treated mice. The reduction in α-synuclein expression specifically in monoamine neurons shall improvise a deficit in dopamine and 5-HT neurotransmission in Parkinson’s disease by enhancing monoamine release and/or reducing uptake. Functional recovery of the nigro-striatal dopamine pathway could be observed upon intranasal administration of IND-1233-ASO in the animal Parkinson’s disease model. Overall, the studies have confirmed the role of non-viral inhibitory oligonucleotides as disease-modifying agents in α-synuclein models of Parkinson’s disease.

### 2.3 MicroRNA

miRNAs are known to possess dynamic functions in regulating biochemical pathways in mammalian brains and hence have been studied as treatment options for several neurogenerative disorders (O’Brien et al., 2018; He and Hannon 2004).

Su et al. (2020) fabricated wheat germ agglutinin modified PEG-PLA nanoparticles loaded with miR132 (WGA-NPs-miR132). miR132 is a micro RNA that is expressed in high amounts in the brain and has great potential in treating Alzheimer’s disease and cerebral ischemia. However, the naked miRNA might undergo easy degradation or excretion in the mucosa post-administration. This warrants the need for a carrier that ensures stability, safety, and targeting ability. Hence, mPEG-PLA was employed as a biodegradable and biocompatible copolymer. Furthermore, the mPEG-PLA nanoparticles were conjugated with lectins to improvise the permeability across the cell membrane and reduce removal by the nasal cilia. The developed nanoparticles were evaluated for particles, size, shape, and morphology. Additionally, the neuroprotective effect of WGA-NPs-miR132 was studied in both murine and rodent models of Alzheimer’s disease with ischemic brain injury and compared with naked-miR132 or NPs-miR132. The developed monodispersed (PDI < 0.25), spherical nanoparticles having a size range of < 200 nm could be majorly transported through olfactory and trigeminal pathways. The biodistribution of WGA-NPs-miR132 in the APP/PS1 mice model and MCAO rats was significantly improved in the brain in comparison to the unmodified nanoparticles, which were majorly distributed in organs such as kidney, liver, and spleen. WGA-NPs-miR132 exhibited better therapeutic activity, reduced nasal ciliary clearance and enhanced targeting to neurons. The authors concluded that intranasal administration of miRNA encapsulating nanoparticles possesses a great potential in the management of neurodegenerative disorders.

Sukumar et al. (2019) developed theranostic polyfunctional gold-iron oxide nanostars containing chemotherapeutic agent (Temozolomide) and surface loaded with therapeutic miRNAs (miR-100 and antimiR-21) for management of glioblastoma. The developmental steps involved the synthesis of gold-iron oxide nanoparticles followed by coating with β-cyclodextrin-chitosan (CD-CS) hybrid polymer, and co-loading with miR-100 and antimiR-21. Further, their surface was decorated with PEG-T7 peptide using the CD-adamantane host-guest chemistry. The nanostars had a size range of 31.3 ± 20 nm, surface potential of −15 mV, and regular spiked surface morphology. Encapsulation of the miRNA within CD-CS coated nanoparticles was found to be 82.89% ± 8.14% miRNA and it provided protection against RNase activity. Similar to the synthetic drug nanoparticles, the release of miRNA from CD-CS-polyGIONs also exhibited a biphasic pattern, wherein the initial phase was characterized by a rapid release and the later phase with a slow and constant release. T7-targeted CD-CS-polyGIONs loaded miRNAs exhibited the highest internalization and intracellular miRNA delivery in comparison to CD-CS-polyGIONs and bare miRNAs when evaluated in human GBM cells (U87-MG). AntimiR-21 when combined with Temozolomide (100 µM), led to a decline in cell viability to 43.8% and miR-100 in combination with Temozolomide (100 µM) led to a decline in cell viability to 54.7 % whereas co-supplementation of both miRNAs (antimiR-21 and miR-100) in combination with Temozolomide (100 µM) led to a further decline in cell viability to 39.2%. The results of this study performed in U87-MG cells indicated the potentiation in Temozolomide anticancer efficacy upon concomitant delivery of both miRNAs as compared to individual administration of miRNAs. The developed nanoplatforms were administered in orthotopic xenograft models in mice *via* the intranasal route. The findings of this *in vivo* study suggested a marked increase in survival of mice (44 days) treated with the targeted nanoparticles in comparison to non-targeted nanoparticles (33 days). Based on the conclusive evidence of *in vitro* cell line and *in vivo* murine model study, the effective therapeutic dose of Temozolomide for glioblastoma could be effectively curtailed by supplementation with therapeutic miRNAs. Thus, this study is a proof of concept exhibiting the merit of this novel theranostic nanoformulation along with the non-invasive nasal route in the management of glioblastoma. Preclinical studies in higher animals, clinical trials, and toxicity evaluation would be conclusive towards the translation of this product from bench to bedside.

Hao et al. (2020) formulated miR-124 containing PEG-PLGA nanoparticles by a double emulsion method. The nanoparticles were surface modified with RVG29, a peptide with 29-amino acid sequence (derived from the rabies virus glycoprotein). RVG29-PEG-PLGA/miRNA-124 nanoparticles were found to possess particle size, PDI, and encapsulation efficiency of 204 nm, 0.4 and 28.2% ± 8.3%, respectively. Sprague Dawley rodent models of middle cerebral artery occlusion (t-MCAO) with ischemic brain injury were employed in the study. Amongst MiR-124, PEG-PLGA/miRNA-124, RVG29-PLGA/miRNA-124, and RVG29-PEG-PLGA/miRNA-124 administered intranasally, RVG29-PEG-PLGA/miRNA-124 nanoparticles exhibited the highest accumulation in the brain. The surface-modified nanoformulation traversed mainly via the olfactory nerve pathway and reached the olfactory bulb region of the brain. The neurological scores of MCAO rats for Sham group, control group, MiR-124, PEG-PLGA/miRNA-124, RVG29-PLGA/miRNA-124, and RVG29-PEG-PLGA/miRNA-124 were found to be 0.00 ± 0.00, 3.50 ± 0.55, 2.83 ± 0.41, 2.67 ± 0.82, 1.83 ± 0.75, and 1.00 ± 0.63, respectively. The beam balance test score scores of MCAO rats for Sham group, control group, MiR-124, PEG-PLGA/miRNA-124, RVG29-PLGA/miRNA-124, and RVG29-PEG-PLGA/miRNA-124 were found to be 5.67 ± 0.52, 1.50 ± 0.84, 2.33 ± 0.52, 3.17 ± 0.75, 4.00 ± 0.63, and 4.83 ± 0.75, respectively. Both these scores are indicative of the excellent therapeutic effect achieved with RVG29-PEG-PLGA/miRNA-124 nanoparticles. RhoA is one of the most abundant scaffold proteins in excitatory neurons and GAP43 is a marker of neurons. RhoA has a negative regulatory effect on neuronal synapse development whereas increased expression of GAP43 is a sign of neural regeneration. MCAO rats were divided into the following treatment groups: RVG29-PEG-PLGA/miRNA-124 nanoparticles, RVG29-PLGA/miRNA-124 nanoparticles, PEG-PLGA/miRNA-124 nanoparticles, miRNA-124, control groups, and sham groups. This was followed by immunofluorescence imaging of the brain to detect the levels of RhoA and GAP43. RVG29-PEG-PLGA/miRNA-124 nanoparticles led to the lowest levels of RhoA and highest levels of GAP43 being expressed amongst all the treatment groups. This could be attributed to the high-affinity interaction of the RVG29 conjugated to the nanoparticle surface with the neuron surface receptors, and the degradation protection offered to miR-124 by the nanoparticles. Thus, the developed nanoplatforms allowed the entry of miRNA to the brain and affected the expression of RhoA and GAP43, which in turn alleviated the symptoms of cerebral ischemia-reperfusion injury. Based on the evidence, the authors concluded that nasal delivery of miR-124 supplemented with RVG29 would contribute to the recovery of neurological function after cerebral ischemia.

Dhaliwal et al. (2020) employed cationic liposomes encapsulating mRNA and studied the potential of the intranasal route in delivery to the brain. The liposomes were prepared by the dry-film formation method and possessed a nanometric size range (195.0 ± 4.5 nm), positive charge (^+^35.6 ± 3.0 mV), monodisperse nature (PDI < 0.2), encapsulation efficiency (80%) and unilamellar characteristics. The transfection efficiency of the GFP-mRNA/Liposomes was tested in J774A.1 murine macrophages. Results exhibited that GFP-mRNA/Liposomes were able to transfect GFP-mRNA in macrophages up to duration of 24 h and stably express GFP protein in the cytosol. *In vivo* biodistribution of GFP-mRNA expression was studied in CD-1 mice by administering GFP-mRNA/Liposomes. GFP-mRNA/Liposomes exhibited significantly higher ∼15% GFP-mRNA expression in the brain in comparison to the naked mRNA group, and vehicle-treated group, at 24 h post-administration. Additionally, the authors concluded that amount of dose; frequency, and time course of protein expression are the vital parameters in governing transfection efficiency in CNS. Thus, the administration of cationic liposomes via the non-invasive nasal route could efficiently deliver mRNA to the specific regions of the brain (cortex, striatum, and mid-brain) with minimal systemic exposure. Thus, intranasal delivery of these nanoplatforms would be crucial in providing therapeutic mRNA treatment in various CNS-related disorders.

### 2.4 Plasmid DNA

Intranasal administration has emerged as a novel non-invasive route for the delivery of plasmid DNA (Khatri et al., 2008).

Park et al. (2002) developed intranasal delivery systems of plasmid DNA employing poloxamers (Pol) as *in situ* gelling polymers and polycarbophil (PC)/polyethylene oxide (PEO) as mucoadhesive polymers. Pol; Pol/PC 0.2%; Pol/PEO 0.4% and Pol/PEO 0.8% were employed as the delivery vehicles for plasmid DNA. All the Pol-based vehicles gelled at the temperature ranges of 31°C–35°C, which is suitable for *in situ* gelling. *In vitro* studies revealed that Pol exhibited the fastest release of plasmid DNA; Pol/PC 0.2% and Pol/PEO 0.4% provide the release of plasmid DNA slower than Pol whereas Pol/PEO 0.8% revealed the slowest release amongst the formulations studied. The kinetic modeling of the *in vitro* release data suggested Fickian behavior. The developed formulations were administered intranasally to male ICR mice and the pharmacokinetic parameters were studied. Based on the AUC values, the formulations were rank-ordered as follows: Pol/PC 0.2% (86519.9 ± 18411.1 pg.h/ml) > Pol/PEO 0.4% (75342.7 ± 13579.1 pg.h/ml) > Pol/PEO 0.8% (33218.4 ± 7,245.6 pg.h/ml). The AUC values of Pol/PC and Pol/PEO 0.4% were 10.9- and 9.6-folds higher than saline, respectively, which could be attributed to the increased contact of plasmid DNA onto the nasal surface in comparison to saline. The levels of plasmid DNA given in Pol/PC 0.2% and Pol/PEO 0.8% were 10- and 40-fold higher relative to the saline vehicle, respectively. Furthermore, Poloxamer-based vehicles might form micelles wrapping the plasmid DNA on the surface of the nasal mucosa and improvise upon the dispersibility and stability of plasmid DNA. When the residual amount of plasmids in the nasal tissues of mice was determined, the vehicles containing *in situ* gelling polymer along with mucoadhesive polymer exhibited much higher retention. The histopathology of the nasal mucosa after repeated dosing did not show any signs of inflammation or tissue necrosis suggesting that Pol/PC or Pol/PEO vehicles could function as safe nasal delivery carriers for plasmid DNA. Overall, the findings of this study indicated that *in situ* gelling and mucoadhesive polymer combinedly could be effective as well as safe in improving the nasal absorption and retention of plasmid DNA. Furthermore, the choice of mucoadhesive polymers and their contents would be the key parameters governing the rate and extent of nasal absorption.

Cui and Mumper (2002) developed novel cationic nanoparticles engineered from a microemulsion precursor. The microemulsion was formed using emulsifying wax as the oil phase. The aqueous phase consisted of cationic surfactant CTAB (hexadecyltrimethyl ammonium bromide)/a combination of CTAB and non-ionic surfactant (Brij 78). Upon simple cooling of the microemulsion to room temperature, nanoparticles were formed. Plasmid DNA was then coated onto the surface of the nanoparticles to obtain pDNA-coated nanoparticles. The *in-vitro* transfection of Hep G2 cells was carried out with the pDNA-coated nanoparticles. The pDNA-coated nanoparticles were administered intranasally to female Balb/C mice. Both types of pDNA-coated nanoparticles [developed using CTAB alone as well as CTAB and Brij 78] increased the transfection efficiency by more than 10-folds in comparison to the naked pDNA. Both types of pDNA-coated nanoparticles exhibited a significant augmentation (18–28 folds) of antigen-specific IgG levels in serum in comparison to naked pDNA. Moreover, the antigen-specific IgA titers in the pDNA-coated nanoparticles immunized mice were elevated by 25- to 30-folds than in comparison to naked pDNA. In addition, an elevated splenocyte proliferative response was recorded after immunization with these pDNA-coated nanoparticles. The results convincingly proved the role of pDNA-coated nanoparticles in enhancing mucosal immunity. Overall, the findings provide concrete evidence about the potential of the developed nanoparticles *via* the intranasal route for the delivery of pDNA.

Oh et al. (2001) studied the biodistribution and extent of absorption of pDNA upon intranasal administration in female BALB/c mice. The maximum serum concentration (Cmax) of plasmids occurred at 90 min, 15 min post-administration, the highest level of plasmids was recorded in the liver whereas cervical lymph nodes recorded the highest level amongst the various lymph nodes. 24 h post-administration, the levels of plasmid DNA in the brain was 3.9- and 4.8-folds higher than that in the lung and the spleen, respectively. The higher distribution of plasmids to the brain after intranasal administration indicates that nasal administration might be a potential route for the delivery of therapeutic genes to the brain with reduced side effects to other organs. Thus, the potential of the non-invasive intranasal route in delivering DNA vaccines and other therapeutic genes could be explored.

### 2.5 Nucleoside analogues

Gemcitabine, a nucleoside analog is a chemotherapeutic drug employed in the treatment of certain solid tumors. Being a small (MW 299.66 daltons) hydrophilic molecule, its transport across the nasal epithelium occurs by paracellular transport (Santoro et al., 2000; De Lange et al., 2004).

In a study carried out by Krishan et al. (2014), gemcitabine was intranasally administered to male Sprague-Dawley rats, wherein the role of papaverine in modulating olfactory tight junctions was evaluated. The histopathology study suggested no histological damage to the olfactory epithelium and the fluorescence study indicated drug delivery to the CNS *via* olfactory epithelium. The Cmax values of gemcitabine for 0% papaverine and 1.4% papaverine treatments in brain extracellular fluid (BECF) were found to be 0.35 ± 0.16 µg/ml and 2.3 ± 1.6 µg/ml, respectively. Similarly, the AUC values of gemcitabine for 0% papaverine and 1.4% papaverine treatments in brain extracellular fluid (BECF) were found to be 1.5 ± 0.28 µg.h/ml and 5.54 ± 0.83 µg.h/ml, respectively. Thus, the Cmax and AUC for gemcitabine in the 1.4% PV treatment group showed a 6.57 and 4-fold increment respectively compared with 0% PV treatment. Upon intranasal administration of gemcitabine + papaverine, the gemcitabine levels in BECF were similar and comparable with those attained by intravenous administration of gemcitabine + BBB permeabilizer (RMP-7). The Tmax achieved with gemcitabine in presence of papaverine and without papaverine was 0.5 h and 1 h, respectively. The study provides a proof of concept that the intranasal route in combination with a non-toxic, reversible epithelial TJ permeabilizer shall provide a useful means of non-invasively delivering nucleoside drugs to the CNS.

5-Fluorouracil, a nucleoside analog has been employed either alone or in synergism with other anticancer drugs to treat various types of malignancies (Miura et al., 2010; Borderwala et al., 2021).

Sakane et al. (1999) studied the brain uptake of 5-FU in Wistar rats following intravenous and nasal application. The concentrations in the cerebral cortex following intravenous infusion, nasal instillation, and nasal perfusion of 5FU were found to be 7,930 ± 438 dpm/g, 12,548 ± 1,094 dpm/g, and 9,094 ± 918 dpm/g, respectively. Similarly, the AUC values following intravenous infusion, nasal instillation, and nasal perfusion of 5-FU were found to be 1,247 ± 41 kdpm.min/ml, 1959 ± 49 kdpm.min/ml, and 1,132 ± 106 kdpm.min/ml, respectively. The CSF/plasma concentration ratio of 5-FU following nasal application was higher than 1 and significantly higher than that following intravenous injection. The results are indicative of the transport of 5-FU from the nasal cavity to the CSF bypassing the BBB.

Shingaki et al. (2009) studied the effect of acetazolamide on the transport and brain uptake of 5-FU in mice upon nasal administration. The control group received 5-FU by the intravenous route. The brain uptake clearance of 5-FU after the nasal perfusion without acetazolamide and with acetazolamide exhibited an increment of 40% and 104% respectively in comparison to intravenous infusion. Thus, the studies confirmed the role of acetazolamide in enhancing the 5-FU delivery to the brain upon intranasal administration.

### 2.6 Apatmers

Aptamers are quite similar to antibodies with respect to specificity and affinity towards targets but work on a broader range of targets in comparison to antibodies. Even though the first aptamer was developed in 1990, it was 15 years down the line that they were used as therapeutic agents. Pegaptanib, with the trade name Macugen, was approved by the United States FDA in 2004 and was the first 28 nucleotide RNA aptamer drug to see the market. There are several documented evidence of aptamers with therapeutic abilities for varying disorders (Bukari et al., 2020; Xiao et al., 2021). Further, aptamers have multiple characteristics that favor their transport across BBB which can be leveraged for delivering therapeutics to the brain (Figure 5). Aptamers can bind with the receptors on the BBB and undergo transcytosis. Additionally, bifunctional aptamers can be used for targeting them to the tumour site.

Multiple research studies have highlighted the abilities of aptamers to transcytose and get internalized in brain endothelial and other similar cell lines. Table 2 depicts the aptamers that have shown potential in traversing across BBB. However, most of the studies are on *in-vitro* models and a few of them have studied the transport process in preclinical models.

**TABLE 2:** Studies on aptamers to show transport across BBB.

Aptamer	*In vitro*/*In vivo* study	Observation	Cellular target	Reference
A15	*In-vivo* in murine model	Transport across BBB	Endothelial Cell	Cheng et al. (2013)
Gint4.T (as aptamer nucleic acid –complex loaded with paclitaxel)	*in vitro* (tumorigenic cell line)	Transcytosis and internalisation	PDGFRB	Shi et al. (2019)
GL21.T (combined with miRNA)	*in vitro*	Transcytosis	Axl	Esposito et al. (2016)
R11-3 and R39	*in vitro*	Internalisation	Human and mouse BBB EC	Dua et al. (2018)
TfRA4 and TEPP (bifunctional aptamer intercalated with doxorubicin)	*in vitro* and *in vivo*	Transcytosis and brain localization	TfR (TfRA4), TfR and EpCAM (TEPP)	Macdonald et al. (2020)

Research studies have confirmed the utility of aptamers in neurodegenerative disorders. The etiology of many neurodegenerative disorders implicates the accumulation of misfolded proteins in the CNS. It is believed that inhibiting the accumulation of these proteins can slow down the overall onset of these diseases. Therefore, aptamers can potentially be useful in the management of several neurodegenerative disorders. Several studies in this line have provided the proof of concept to the notion of the use of RNA aptamers (N2 and E2) that bind to Aβ1–40, RNA aptamer (N2) ruthenium complex system to inhibit Aβ aggregation, M5–15, F5R1, and F5R2 aptamers against α-syn oligomers. However, systematic studies exploring aptamer delivery through the nasal route are yet to be seen and are a potential area for research (Cheng et al., 2013; Dua et al., 2018; Sanchez-Ramos et al., 2018; Bukari et al., 2020; Xiao et al., 2021).

NBTs are either small molecule or macromolecular agents, that have been employed in the management of several disorders as mentioned in Section 1.2. However effective these may be, they do have long term or delayed onset side effects. There is a need to develop strategies to overcome the toxicity aspect of NBTs so that their clinical acceptability increases.

## 3 Toxicity of Nucleic acid based therapeutics and strategies to overcome them

The undesirable effects associated with the delivery of small molecule NBTs can be classified into both hematological and immunological types. These effects can be further detailed as follows:

1) Hematological: e.g., anemia and thrombosis (can be life-threatening)
2) Immunological: Further classified as

Immunostimulatory/immunomodulatory: e.g., autoimmune hemolytic anemia, anaphylaxis, cytokine release syndrome, delayed-type hypersensitivity reactions, and systemic inflammatory response syndrome reactions.

1) Immunosuppressive: e.g., thrombocytopenia, myelo-suppression, neutropenia, lympho-adenopathy, and suppression of immune response to common antigens.

The undesirable effects associated with delivery of macro molecular NBTs can also be classified into both hematological and immunological types. These effects can be further detailed as follows:

1) Hematological: prolongation of plasma coagulation time, complement activation, and a variety of hematological changes such as leukopenia, anemia, thrombocytopenia, and lymphopenia.
2) Immunological: The majority of side effects in this category are of immunostimulatory/immunomodulatory type. They include induction of (a) cytokines, (b) chemokines, (c) type I and type II interferons, (d) fever, and (e) activation of monocytes, T cells, B cells, NK cells and dendritic cells.

Based on the previous preclinical and clinical translational studies, it was been found that immunostimulation occurs majorly due to TLRs (TLR3, TLR7/8, and TLR9). However, recent studies have suggested that in addition to TLRs, several other proteins directly bind to DNA or RNA and initiate potent immune responses. One of the strategies that would help in overcoming the translational challenges arising from the immunostimulatory properties of NBTs includes the use of delivery vehicles, which play a vital role in masking NBTs from the immune surveillance system. Amongst the delivery vehicles, nanocarriers have received much attention in recent times. In addition to the use of nanoplatforms, the following approaches have been considered by the researchers 1) modifying the dose and dosing schedule to keep NAT plasma concentrations at sub-threshold levels, 2) co-administration with cytotoxic oncology drugs, 3) co-loading nanoparticles with immunosuppressive reagents, 4) pre-medicating patients with an anti-pyretic, anti-histamine, and immunosuppressive drug cocktail (iv) application of a-CD3 and a-CD20 antibodies to deplete circulating B cells (Dobrovolskaia and McNeil, 2015a; Esposito et al., 2016).

### 3.1 Choosing a nanocarrier

Nanoparticles are known to increase the NAT concentration of the encapsulated NAT in the CSF or brain tissue upon intranasal administration as compared to the same drug administered as a simple solution. While choosing a nanocarrier for NATs, due consideration must be given to its size, charge, and hydrophobicity. These physicochemical properties must be tuned to improvise the biopharmaceutical and pharmacokinetic properties and to reduce or eliminate undesirable effects. In a study carried out by Yadav et al. (2016), the authors had laid down a constraint of < 400 nm on the particle size of the cationic nanoemulsion developed. The smaller particle size shall in turn provide a higher surface area for interaction and absorption in the olfactory epithelium. Additionally, greater particle surface area shall prevent immune recognition in the bloodstream and increase particle circulation time for maximal delivery to the target site.

The nasal delivery offers a very short residence time for molecules; wherein the half-life for clearance for non-adhesive liquid and powder type formulations is of the order of 15–30 min. Perez et al. (2012) employed chitosan (1% w/w) or Carbopol (0.25% w/w) as mucoadhesive agents while formulating Poloxamer based *in situ* mucoadhesive gels. Initially, siRNA dendriplexes were formed by electrostatic complexation of siRNA with cationic dendrimers, which were then incorporated into the gels. The dendriplexes exhibited reduced degradation by RNAses and increased endocytic uptake. Poloxamer employed in this study is a free-flowing liquid at room temperature and gets converted to a gel-like consistency at physiological temperatures. The use of mucoadhesive polymers supplements the developed nanoplatforms by increasing the time of residence of the loaded material.

In a study carried out by Sava et al. (2020), chitosan was employed as a matrix-forming polymer of the nanoparticles. Here, electrostatic interactions were found to occur between the positively charged moieties of the amino groups of Chitosan and negatively charged phosphate moieties of the siRNA structure. Additionally, Mangafodipir was employed as a cross-linking agent in the nanoparticles to stabilize the globular structure of NP and protect siRNA from degradation. The divalent metal transporter might be crucial in the early endosomal processing of the NPs.

In another study, Azambuja et al. (2020) employed cationic nanoemulsion due to its ability to form complexes with the negatively charged siRNAs, allowing cell uptake and consequent interaction with the intracellular target of siRNA sequences.

Rassu et al. (2017) prepared BACE1 siRNA complex with a cell-penetrating peptide (RVG-9R) and loaded it in solid lipid nanoparticles, which were further coated with chitosan. RVG-9R was found to protect the oligonucleotide and enhance the intracellular pathway by receptor-mediated endocytosis within the neurons. The coating process provided siRNA protection, ensured mucoadhesiveness to the particles, and also prolonged residence time in the nasal cavity. siRNA was found to permeate the Caco-2 monolayer to a greater extent when released from chitosan-coated SLNs in comparison to uncoated SLNs and bare siRNA.

Thus, choosing a nanocarrier for NATs would include careful evaluation of all precursors and components of nanoformulation and assessing their immunological/hematological reactivity. The extent of change in the physicochemical parameters, stability, uptake, biodistribution, and PK parameters of NATs due to the nanocarriers also needs to be considered (Dobrovolskaia M,A. et al, 2015).

## 4 Regulatory challenges

Nucleic acid-based therapeutics marks a new generation of therapeutic agents employed for a wide array of disorders including CNS disorders. In the last 1–2 decades, the number of NBTs that are approved or undergoing clinical trials is on the rise. The first in the category to be approved was pegatinib (Watts et al., 2019). Table 3 below provides information about these products.

**TABLE 3:** Approved/under trial NBTs for CNS disorders.

Drug	Category	Disease	Year of approval	Administration
Pegaptanib	RNA aptamer	Age-related macular degeneration	2004	Intravitreal
Nusinersen	SS antisense RNA (Splice-switching ASO)	Spinal muscular atrophy	2016	Intrathecal
Eteplirsen	SS antisense RNA (Splice-switching ASO)	Duchene muscular dystrophy	2016	Intravenous
Inotersen	SS antisense RNA (Gapmer ASO)	Hereditary transthyretin amyloidosis	2018	Subcutaneous
Patisiran	DS siRNA	Hereditary transthyretin amyloidosis	2018	Intravenous
Golodirsen	SS antisense RNA (Splice-switching ASO)	Duchene muscular dystrophy	2019	Intravenous
Onasemnogene abeparvovec	Delivery of SMN (gene therapy)	Spinal muscular atrophy	2019	Intravenous
Viltolarsen	Antisense RNA	Duchenne muscular dystrophy	2020	Intravenous
Casimersen	Antisense RNA (steric block ASO)	Duchenne muscular dystrophy	2021	Intravenous
Tofersen	ASO	Amyotrophic Lateral Sclerosis	Under trial	Intrathecal
Jacifusen	ASO	Amyotrophic Lateral Sclerosis	Under trial	Intrathecal
BIIB100	ASO	Amyotrophic Lateral Sclerosis	Under trial	Intrathecal
BIIB094	ASO	Parkinsons disease	Under trial	Intrathecal
ALT1102	ASO	Multiple Sclerosis	Under trial	Subcutaneous
AMT-130	miRNA/AAV5	Huntington’s diseases	Under trial	IST
ION373	ASO	Alexander disease	Under trial	Intrathecal

Regulatory agencies such as the USFDA/EMA have guidelines or draft guidelines in place about the approval of biological products and gene therapy products. These guidelines discuss the need for proper choice of animal strain, the number of animals to be included in the study, duration of the study, and biodistribution study of the nucleic acid/excipients to justify the outcomes, and translation to clinical settings. The major issues faced by pharmaceutical companies in the regulatory approval of NBTs include specificity, tolerability, and toxicity. Further, the different classes of NBTs differ in their composition, configuration, carrier employed, and route of administration, making the regulatory process complex (Food and Drug Administration, 2013; Alarcón-Arís et al., 2018; European Medicines Agency, 2018; Food and Drug Administration, 2020; ICH, 2021; Vervaeke et al., 2022). In this review, we have focussed on the nasal route for delivering nucleic acid therapeutics. Although investigations exploring this non-invasive route for nucleic acid therapeutics are steadily on the rise, none of the products are approved for clinical use through the nasal route. The challenges about nucleic acid therapeutics and the nasal route have been described above.

The third aspect of regulatory perspectives in nucleic acid therapeutics is the regulatory framework concerning nanomaterials. More than 50 nanotechnology-based products are approved for marketing. Yet specific and harmonized regulations about nanomedicines are still missing. Starting from design to its marketing, nanomedicines are under the purview of regulatory eyes to ensure that the efficacy, quality, and safety of the product are not compromised. Different regulatory agencies have floated draft guidance in the last few years. But inconsistencies galore across the globe as some agencies treat nanomedicines as therapeutics while others deem them as medical devices. There are several challenges in the development of regulatory guidance for such products which have hindered the translation of explicit guidelines. The variable morphology of nanomedicines, unusual pharmacokinetics, drastically different permeation, and accumulation characteristics, scale-up technicalities, stability issues, toxicity *in-vivo* as well as the impact on the environment have been the major issues of contention. Despite the lack of uniformity, the number of nanotech-based products on the shelf is steadily increasing due to improved characterization techniques. Nucleic acid therapeutics also face these challenges of nanotechnology as they are rarely delivered naked and are enclosed in nanostructures before administration (Dobrovolskaia and McNeil, 2015b; Foulkes et al., 2020; Souto et al., 2020).

The coming years are going to be defining for this therapeutic category, which is still nascent. An efficient and encompassing regulatory framework addressing the stability, toxicological concerns, delivery or internalization aspects, and above all specificity criticalities will enable more nucleic acid therapeutics into the market.

## 5 Future perspective

It has been around 40 years since the plausibility of nucleic acid-based therapeutic was first explored. The studies in the first two decades were more focused on exploration and development with limited clinical translation. The last two decades have made these molecules a practical possibility. The upcoming two decades will define and unleash the potential of this truly remarkable therapeutics. These molecules have opened the vistas for the management and treatment of various hereditary and complex disorders. These moieties possess nearly unlimited capacity to address unmet clinical needs for several diseases including CNS-based disorders. The journey so far has not been an easy ride because of the specificity, tolerability, and delivery issues of nucleic acid therapeutics. The advances in diagnostics and preclinical areas about the field have given the impetus to a successful translation. Advanced delivery technologies and bio-conjugation of the molecules have made it possible to overcome the limitations of reaching the target sites in desirable concentrations. These have also aided in suitably manipulating the pharmacokinetics as well.

One potential play area for nucleic therapeutics is in the field of CNS disorders, but access to the targets in CNS is challenging. This has propelled the investigations into the intranasal delivery of such therapeutics. Direct nose-to-brain delivery is extremely promising but at the same time highly variable and unpredictable. The nasal route offers several merits of non-invasiveness, patient compliance, and improved bioabsorption. Hence, the number of nucleic acid-based drugs under development, and in clinical trials to be delivered by nasal route, is growing rapidly. Systematic studies in this direction are giving positive indications for possible clinical translation. However, to date, none of the NBTs by nasal route has been able to be translated from the bench to bedside. Despite the research focus, there are several grappling issues to be addressed before we see more intranasally delivered nucleic acid therapeutics getting the regulatory nod. The stability, specificity, tolerability, and delivery concerns are being addressed. Although there is room for further improvement and innovation in each of these areas, the solutions have advanced to the stage that nasal delivery of nucleic acid-based therapeutics would soon be employed in a clinic setting.

The strides made in the areas of chemical modification, backbone modification, stereochemistry, nucleobase modification, terminal modification, ribose sugar modification, etc., have been exemplary. The chemical modifications could lead to increased potency; altered pharmacokinetics, pharmacodynamics, and biodistribution properties; and potentially decreased toxicological liabilities whereas improved backbone modification would confer nuclease resistance; promote binding to proteins in both plasma and within cells; prolong tissue retention and provide long-lasting drug effects. In the future, the upgraded knowledge of stereochemistry and nucleobase modification would help in synthesizing nucleic acid therapeutics with defined stereochemistry and providing targeting ability/specificity, respectively. Advancement in the knowledge about lipids, peptides, aptamers, antibodies, and sugars would help in improving bio-conjugation of nucleic acid therapeutics with these moieties, which shall in turn promote intracellular uptake, targeting specific cells/tissues or reduce clearance from the circulation.

Of the explored nanovectors, non-viral nanoplatform delivery technologies like lipid nanoparticles are grabbing the attention and trust of researchers. The upcoming developments in the fields of biodegradable/biocompatible polymers, material science, and nanoplatforms would help in addressing the delivery challenges such as crossing biological barriers and transmembrane intracellular delivery. Optimization of nanoplatforms in terms of biophysical (size, shape, and chemical/material composition) and biological (ligand functionalization for targeting) attributes shall allow the development of highly tailored delivery platforms. With the synergism of native and chemically modified nucleic acid therapeutics along with powerful and versatile nanoplatforms, nucleic acid-based therapeutics are destined to change the standard of care for many diseases including CNS disorders.

The advanced gene editing platforms, chemical and functional modifications like bio-conjugation strategies, leveraging nanovectors, and functionalizing them to be stimuli sensitive are going to be the driving factors in the future. The coming years will witness the development of specific and targeted delivery of nucleic acid therapeutics through the nasal route for better therapeutic outcomes.
